# Involvement of Male Partners of Pregnant Women in the Prevention of Mother-to-Child Transmission (PMTCT) of HIV in Haiti: A Mixed-Methods Study

**DOI:** 10.1177/15579883211006003

**Published:** 2021-04-19

**Authors:** Patrice Ngangue, Middle Fleurantin, Rheda Adekpedjou, Leonel Philibert, Marie-Pierre Gagnon

**Affiliations:** 1 Faculté de Médecine et des Sciences de la Santé, Université de Sherbrooke, Sherbrooke, QC, Canada; 2 Faculté de Médecine, Université Laval, Québec, QC, Canada; 3 Faculté des Sciences Infirmières, Université Laval, Québec, QC, Canada

**Keywords:** Male involvement, HIV/AIDS, mother-to-child transmission, gender role, mixed-methods

## Abstract

This mixed-methods study aimed to determine the level of male involvement in the prevention of mother-to-child transmission (PMTCT) services in Haiti and identify barriers and associated factors. From May to June 2018, a questionnaire was used to measure the level of male involvement. Semistructured interviews with pregnant women were also conducted. Multivariate linear regression and qualitative content analyses were performed to explore factors associated and barriers to male partners’ involvement in PMTCT services. One hundred and two pregnant women living with HIV completed the questionnaire. About 47% of male partners had a high level of involvement. Specifically, 90% financially supported their spouse, and 82% knew her appointment date at the antenatal clinic (ANC). Only 25% of male partners accompanied their spouse to the ANC, and 19% routinely used a condom during sexual intercourse. Factors associated with male involvement in PMTCT were being married and sharing HIV status with the male partner. Male partners with a positive HIV status were more likely to be involved in PMTCT. Qualitative findings revealed that barriers to male involvement included the conflict between opening hours of the ANC and the male partner’s schedule, waiting time at the ANC, and the perception of antenatal care as being women’s business. Overall male partners’ involvement in PMTCT services is moderate. Gender relations, sociocultural beliefs, and care organization are likely to hinder this involvement. Developing and implementing contextually and culturally accepted strategies for male partners of pregnant women could contribute to strengthening their involvement in the PMTCT program.

## Background

In 2018, there were 1.7 million children (<15 years) living with HIV worldwide (UNAIDS, 2019). Mother-to-child transmission (MTCT) of HIV is the main route through which children are infected (World Health Organization (WHO), 2010). About 86% of the estimated 160,000 children newly infected with HIV in 2018 were in the WHO African Region (World Health Organization (WHO), 2019), mostly because of inadequate access to HIV prevention, care, and treatment services (UNICEF, 2019). Studies have reported that the risk of a woman living with HIV transmitting the virus to her child can be reduced to less than 2% with effective antiretroviral therapy (ART) during pregnancy, childbirth, and breastfeeding periods (Chinkonde et al., 2009; Moodley et al., 2009). The prevention of new HIV infections coupled with early access to antenatal care and HIV testing is vital to the PMTCT (Bras et al., 2015).

While ensuring better coverage of ART for pregnant women, numerous systematic reviews interventions targeting factors that may influence their use of prevention of mother-to-child transmission (PMTCT) services are complementary to help reduce MTCT more quickly (Ambia & Mandala, 2016; Mutabazi et al., 2017; Organisation mondiale de la santé (OMS), 2013; Wettstein et al., 2012). Among these interventions, the involvement of pregnant women’s partners in antenatal care and PMTCT has identified that it can improve outcomes of PMTCT programs in resource-limited settings such as uptake and adherence to ART during pregnancy and delivery, enhancement of HIV-free survival among children (Dunlap et al., 2014; Frederick Morfaw et al., 2013; Manjate Cuco et al., 2015; Organisation mondiale de la santé (OMS), 2012; Takah et al., 2018).

According to the World Health Organization (WHO) (Organisation mondiale de la santé [OMS], 2012), men’s involvement in PMTCT is defined by the presence of men in the prenatal counseling and testing for HIV and behavioral and health outcomes associated with them (e.g., condom use, acceptance of HIV testing during pregnancy, and participation of men in the birth preparation plan). A partner is considered to be involved in PMTCT services if he: (1) accompanies his partner in prenatal visits; (2) knows the prenatal appointments of his partner; (3) discusses prenatal interventions with his partner; (4) takes the time to discuss what is going on in prenatal consultations; (5) participates in financing the monitoring of the care of his partner during the prenatal and postnatal period; and (6) uses a condom during sexual intercourse with a partner during pregnancy (Amano & Musa, 2016; Byamugisha et al., 2010).

Men play an essential role in preventing and transmitting infection regarding condom use in the couple (Desgrees-du-Lou et al., 2009; Mullany et al., 2007). When men are more involved in PMTCT, there is better adherence to ART among their spouses (Fatch et al., 2013; Robert Byamugisha, et al. 2010) and a decrease in MTCT. The male partner influences women’s decisions about newborn care, including accepting and giving medications and following advice on eating (Msuya et al., 2008; Mullany et al., 2007). Conversely, the male partner support could imbalance power dynamics and decision-making within the couple (Hampanda et al., 2020). This could negatively affect women’s PMTCT-related health behaviors, such as ART uptake (Hampanda et al., 2020).

Despite all the benefits of involving men in PMTCT, these programs have mostly focused on women and excluded their male partners, especially in resource-limited settings (Morfaw et al., 2013). Women do not manage and take decisions about their pregnancy alone. PMTCT sites should have adequate space, flexible hours of operation, and consistent policies and regulations to accommodate expectant mothers and their spouses/partners (Dunlap et al., 2014; Reece et al., 2010).

In Haiti, HIV infection is a significant public health problem. According to UNAIDS, 2.0% of the adult population was infected with HIV in 2018 (Ministère de la santé publique et de la population (MSPP), 2012). In 2015, the National AIDS Program (PNLS) reported a rate of MTCT of 7.1% (Monitoring, 2015). During the same period, 85% of pregnant women attending prenatal consultations were tested for HIV. About 90% of HIV-positive women and newborn babies received ART to prevent MTCT (Monitoring, 2015). Despite these findings among pregnant women and their children, the number of partners of HIV-infected pregnant women who have benefited from any service aimed at preventing MTCT is not well known by the main actors of the PNLS in Haiti.

The reason is that PMTCT services focus exclusively on pregnant women, ignoring the place of male partners in the mechanisms of MTCT of HIV (Morfaw et al., 2013). Because of the benefits of male involvement in improving the PMTCT program outcomes and the influence of Haitian men on women’s health decisions, particularly on pregnancy services (Pierre & Maitre, 2010), research into this issue is needed.

To the best of our knowledge, no study has been conducted in the Haitian context on this subject. This study aimed to (1) determine the level of involvement of male partners of pregnant women living with HIV in PMTCT services, particularly prenatal services; and (2) identify associated factors and barriers to their involvement in PMTCT.

## Methods

### Study Setting and Design

The study took place in two health facilities of the Saint-Marc health subdivision unit (HSU) in the Artibonite Department. These are Dumarsais Estimé health centers in Verrettes, and first-line health service (SSPE) in Saint-Marc. According to the report of the preliminary results of EMMUS-VI, the Artibonite Department reported a prevalence of 2.7% among adults, the highest in the country. Besides, the St. Marc HSU had the highest proportion of pregnant women living with HIV monitored by the National AIDS Program, 35% in 2016 (Monitoring, 2015). The two sites were selected using purposive sampling.

A concurrent mixed-method cross-sectional study was conducted among pregnant women living with HIV, monitored in antenatal clinics (ANCs) at Dumarsais Estimé in Verrettes and SSPE in Saint-Marc.

### Study Population and Sampling

The study population consisted of pregnant women living with HIV followed by ANCs as part of the PMTCT. A convenience sample was used to recruit study participants. Pregnant women seen in prenatal care at any ANC visit were invited to sign an informed consent form to participate in the study following the presentation of the study objectives and the PMTCT nurse’s explanation. Thus, all HIV-infected pregnant women were approached to participate in the study as they presented themselves at the ANC. PMTCT nurses ensured the recruitment of participants. For the qualitative part, purposive sampling was used. Every pregnant woman who filled the questionnaire was invited by a PMTCT nurse to voluntarily participate in a semistructured interview. We intended to select 12 participants. The interviews were conducted in creole by one research team member (MF).

A total of 102 HIV-infected pregnant women were recruited to participate in the study, and six of them participated in semistructured individual interviews. Data were collected after their ANC appointment.

### Level of Male Involvement in PMTCT

In the present study, the level of male involvement in PMTCT was determined using a male involvement index. This index was previously used in two studies conducted in Uganda (Byamugisha et al., 2010) and Ethiopia (Amano & Musa, 2016). To be considered involved or not, the index variables were counted according to a score of equal weight. A rating of 0 is assigned to an answer “No” = no involvement and a rating of 1 to any answer “Yes” = involvement (e.g., “Ever attended ANC with a partner,” “knows partner’s ANC appointments,” “provides financial support to partner to attend ANC”. . .). Therefore, the index score ranged from 0 to 6. A total score of 4–6 was considered a “high male involvement” score and 0–3 as a “low male involvement” score.

### Data Collection and Analyses

Data collection took place from May to June 2018. The quantitative and qualitative data have been collected at the same time. For the quantitative component, pregnant women recruited in the study were administered the male involvement index questionnaire by PMTCT nurses. The questionnaire was pre-tested with two pregnant women living with HIV followed in another health center. In addition to the male involvement questions, the questionnaire was also used to collect male partners sociodemographic characteristics (age, marital status, place of residence, occupation, education level) and their history of HIV testing (partner’s HIV status, sharing status between partners). The response format was yes/no (dichotomous). The questionnaire was administered to pregnant women by the PMTCT nurses, and the average filling time was 25 min.

Descriptive statistics were performed to describe the characteristics of the pregnant women, male partners’ characteristics as reported by the pregnant women, and the level of involvement of partners in PMTCT services. To explore factors associated with male partners’ involvement in PMTCT services (dependent variable), bivariate linear regression analyses were performed to examine the relationship between characteristics of pregnant women, characteristics of male partners, and the index score of male partners’ involvement (at 0.20 alpha level) (Maldonado & Greenland, 1993). Following bivariate analyses, multivariate linear regression analyses using backward elimination for model selection were performed. Two models were computed: in one model, the level of involvement of partners was regressed on characteristics of pregnant women; in the other model, the level of involvement of partners was regressed on characteristics of male partners as reported by the pregnant women. Due to the analysis’s exploratory nature, a *p* value of <.10 was used as the threshold for statistical significance in the final models.

### Qualitative Data Collection and Analysis

For the qualitative part, pregnant women were invited to share the reasons that might or might not encourage their partner to become involved in prenatal services during a semistructured interview. All interviews were conducted using a semistructured guide. The data from the literature provided the framework for the development of the interview guide (Morfaw et al., 2013; Yeganeh et al., 2017). The average length of the interviews was 25–30 min. Interviews were digitally recorded and transcribed verbatim by a trained transcriptionist and cleaned for accuracy by a research assistant. Directed qualitative content analysis was performed using mixed coding (Schreier, 2017). Six steps of content analysis were followed through the process.

One team research member (MF) reads through all transcripts and identified possible themes (step 1). Two team members (MF and PN) developed a coding scheme inductively from the data based on an independent review of three transcripts. Discussions with the research team reached agreement on a final coding scheme. Two people independently (MF and PN) used this to code all transcripts using NVivo V.11.0 to assist with data management (step 2). Together, the two team members discussed and identified recurring and converging codes across participants. The recurring and converging codes were then grouped in categories. The categorization was discussed and agreed upon with other research team members (steps 3 and 4). Finally, each category (barrier) was named, defined, and a written report generated. Key quotes that illustrated each category (barrier) were extrapolated from the data (steps 5 and 6). As a result, the main barriers influencing male involvement in PMTCT were identified. NVivo version 11.0 software (QSR International Pty Ltd) was used for the analysis.

The credibility was enhanced by developing a prior familiarity with the participants’ culture before the process of data collection commenced (Lincoln & Guba, 1985). Interview sessions were cross-checked to understand the data by playing back the recorded data to them for corrections. Members of the research team reviewed the data to ensure that the results were consistent. The quantitative and qualitative data were analyzed separately and were integrated during the interpretation of the results.

The quantitative and qualitative findings were discussed among the authors to achieve an understanding of the male involvement in PMCTC. The reliability of the analysis was ensured through data triangulation and peer debriefing during regular team meetings.

### Ethical Approval

The study received an agreement of the executive director of the two health institutions involved in the study. Participants were invited to participate in the project voluntarily. All the participants gave their written consent after knowing the purpose, the nature of the study, and the importance of their participation. In the absence of an ethics committee that well functions in Haiti, this research was approved by the Monitoring-Evaluation and Research service of the National Program against HIV/AIDS (Number PNLS/1926/2018).

### Data Availability Statement

The data that support the findings of this study are available on request from the corresponding author. The data are not publicly available due to privacy or ethical restrictions.

## Results

### Quantitative Results

Of the 128 pregnant women living with HIV who were approached to participate in the ANC study, 102 of them gave their consent and were interviewed at the two sites. The participation rate was 79.68%.

The characteristics of pregnant women living with HIV who participated in the study are presented in Table 1.

**Table 1.** Characteristics of Pregnant Women (*n* = 102).

Variables	*n* (%)
**Age (years)** Mean (*SD*)	27.52 (5.89)
**Number of children** Mean (*SD*)	1 (2)
**Gestational age (weeks)** Mean (*SD*)	21 (8)
**Number of antenatal consultations** Mean (*SD*)	2 (1)
**Religion**	
Catholic	18 (17.65)
Protestant	58 (56.86)
Voodoo	2 (1.96)
Others	3 (2.94)
No religion	21 (20.59)
**Setting**	
Rural	63 (61.77)
Urban	39 (38.23)
**Marital status**	
Married	17 (16.67)
Free union	63 (61.76)
Single	22 (21.57)
**Education level**	
Primary	32 (31.37)
Secondary	36 (35.29)
University	4 (3.92)
Do not know how to read	30 (29.41)
**Occupation**	
Agriculture	8 (7.84)
Qualified worker	7 (6.86)
Non-qualified worker	1 (0.98)
Small business	57 (55.88)
Housewife	1 (0.98)
Unemployed	28 (27.45)
**Shared her HIV status with her partner**	
Yes	28 (27.45)
No	74 (72.55)

#### Sociodemographic Characteristics of Male Partners Reported by Pregnant Women

The average age of male partners was 35 years (±7.19). The majority were Protestant (*n* = 51; 50%), followed by Catholics (*n* = 22; 21.57%) and no religion (*n* = 22; 21.57%). As with pregnant women, a higher proportion of male partners were living in rural areas (*n* = 63; 61.77%). The majority of male partners were in a free union (*n* = 63; 62%). More than 55% of them attended secondary school, while 34.31% (*n* = 35) had a primary level. In terms of occupation, 41.17% (*n* = 42) of male partners managed a small business, and the rest was shared between those who were unskilled workers (*n* = 25; 24.50%) and those who worked in the agricultural sector (*n* = 17; 16.67%) (Table 2).

**Table 2.** Characteristics of Male Partners as Reported by Women (*n* = 102).

Variables	*n* (%)
**Age (years)** Mean (*SD*)	35.55 (7.19)
**Religion**	
Catholic	22 (21.57)
Protestant	51 (50)
Vodouisant	7 (6.86)
No religion	22 (21.57)
**Setting**	
Rural	63 (61.77)
Urban	39 (38.23)
**Marital status**	
Married	17 (16.67)
Free union	63 (61.76)
Single	22 (21.57)
**Education level**	
Primary	35 (34.31)
Secondary	57 (55.89)
University	10 (9.80)
**Occupation**	
Agriculture	17 (16.67)
Qualified worker	12 (11.77)
Small business	42 (41.17)
Unskilled worker	25 (24.50)
Unemployed	6 (5.88)
**HIV status**	
Positive	11 (10.78)
Negative	21 (20.59)
Do not know	70 (68.63)

#### Level of Involvement of Male Partners in the PMTCT Program and Factors Associated With Involvement

The level of involvement of male partners in PMTCT was calculated using six variables (Table 3). Analysis of the index of male partner involvement in the PMTCT program showed that 52 (50.98%) partners scored high on the male involvement index. Regarding the dimensions of the involvement, most pregnant women (*n* = 92; 90.20%) reported that their partners supported them financially for purchasing medicines or for the payment of transport costs to go to the ANC. A large proportion of the pregnant women (*n* = 84; 82.35%) revealed that their partners knew the appointment date of their ANC visit. A large proportion (*n* = 74; 72.55%) of the partners also discussed the pregnancy’s monitoring with their female partner, such as the delivery. About 60.78% (*n* = 62) of the partners were interested to know what happened during the ANC visit. In contrast, only a quarter of male partners (*n* = 26; 25.5%) accompanied their women to the ANC, and an even smaller proportion (*n* = 19; 18.63%) routinely used a condom during pregnancy.

**Table 3.** Level of Involvement of Male Partners in PMTCT Services.

Dumarsais Estimé and SSPE of St-Marc (*N* = 102)		
Dimensions of male involvement in PMTCT	Answers *n* (%)	
	Yes	No
Your partner accompanies you to past ANC or ANC today	26 (25.5)	76 (74.5)
Your partner gives you the cost of transportation or pays for your medication	92 (90.20)	10 (9.80)
Your partner discusses prenatal interventions with you	74 (72.55)	28 (27.45)
Your partner took the trouble to find out what happened during antenatal consultations	62 (60.78)	40 (39.22)
Your partner knew your appointment today in ANC	84 (82.35)	18 (17.65)
Your partner always uses a condom during sex with you during your pregnancy	19 (18.63)	83 (81.37)
Level of involvement of partners*	**High**	**Low**
	52 (50.98)	50 (49.02)
Mean involvement level of partners (*SD*)**	3.50 (1.27)	

*Note*. *A total score of 4 to 6 was considered a “high” male involvement score and 0 to 3 as a “low involvement” score.

** Level of involvement ranges from 0 (no involvement) to 6 (high involvement).

PMTCT = prevention of mother-to-child transmission; ANC = antenatal clinic.

#### Factors Associated With Male Partner Involvement in PMTCT

In multivariate linear regression analysis regressing level of involvement of male partners on pregnant women characteristics, being married was associated with higher male partners’ involvement compared to being in a free union (β = 0.78, *p* = .022) and sharing pregnant women’s HIV status with her partner was associated with higher male partners’ involvement compared to not sharing (β = 0.64, *p* = .022) (Table 4).

**Table 4.** Level of Involvement of Male Partners in PMTCT Services Regressed on Pregnant Women Characteristics (*n* = 102).

Variables	Bivariate Analyses			Multivariate Analyses		
	*Β*	95% CI	*p* value	β	95% CI	*p* value
**Age** (years)	0.006	[–0.037, 0.049]	.782	–	–	–
**Number of children**	0.09	[–0.06, 0.24]	.255			
**Gestational age** (weeks)	0.01	[–0.01, 0.04]	.350			
**Number of antenatal consultations**	0.03	[–0.19, 0.26]	.753			
**Religion**	0.990					
Catholic (vs. no religion)	−0.09	[–0.91, 0.74]	.834	–	–	–
Protestant (vs. no religion)	0.07	[–0.58, 0.73]	.819	–	–	–
Voodoo (vs. no religion)	0.02	[–1.88, 1.92]	.980	–	–	–
Others (vs. no religion)	−0.14	[–1.73, 1.44]	.858	–	–	–
**Setting**						
Urban (vs. rural)	0.02	[–0.49, 0.53]	.936	–	–	–
**Marital status**	**0.045**			**0.066**		
Single (vs. free union)	0.18	[–0.43, 0.79]	.555	0.28	[–0.32, 0.88]	.363
Married (vs. free union)	0.86	[0.18, 1.53]	.013	0.78	[0.11, 1.44]	.022
**Education level**	**0.101**					
Primary (vs. do not know how to read)	0.43	[–0.19, 1.06]	.176	–	–	–
Secondary (vs. do not know how to read)	0.74	[0.12, 1.35]	.018	–	–	–
University (vs. do not know how to read)	0.93	[–0.40, 2.25]	.164	–	–	–
**Occupation**	**0.078**					
Agriculture (vs. no unemployed)	−1.05	[–2.04, –0.06]	.036	–	–	–
Qualified worker (vs. no unemployed)	0.14	[–0.89, 1.18]	.785	–	–	–
Non-qualified worker (vs. no unemployed)	1.57	[–0.93, 4.07]	.216	–	–	–
Small business (vs. no unemployed)	0.20	[–0.36, 0.77]	.479	–	–	–
Housewife (vs. no unemployed)	1.57	[–0.93, 4.07]	.216			
**Shared her HIV status with her partner**						
Yes (vs. no)	0.69	[0.14, 1.23]	**.013**	0.64	[0.09, 1.19]	**.022**

*Note*. “-” indicates that this variable was not kept in the final model. PMTCT = prevention of mother-to-child transmission.

In multivariate linear regression analysis regressing level of involvement of male partners on male partners’ characteristics, being a qualified worker, a non-qualified worker, and doing a small business were associated with higher male involvement compared to being unemployed (β = 1.26, *p* = .036; β = 1.20, *p* = .027; β = 1.50, *p* = .004, respectively). Knowing his HIV status, whether positive or negative, was associated with higher male involvement than not knowing (β = 1.12, *p* = .004 for positive HIV status; β = 0.67, *p* = .028 for negative HIV status) (Table 4).

## Qualitative Findings

A total of six pregnant women participated in semistructured interviews. The participants mentioned several factors that may prevent partners of pregnant women from participating in the PMTCT program. These multiple reasons were related to partners, gender relations between men and women, cultural beliefs, and the organization of PMTCT care and services.

### Barriers to Male Involvement in PMTCT

When pregnant women were to share their opinions on the reasons that may prevent their partners from accompanying them to the ANC, men’s lack of time to come to the ANC was the main reason identified. Since male partners have to work to meet the family’s needs, they do not have time to attend the ANC. Also, the opening hours of the health centers are incompatible with men’s work schedules, as illustrated in this excerpt:

My husband works for six days a week, and in most cases, he leaves at 6:00 am and returns late in the evening [. . .]. PMTCT services are closed on weekends and at 4 pm during the week. My husband goes to work instead of coming to the hospital. (pregnant woman 02)

Another barrier reported by respondents is that men traditionally view maternal health services like women’s affairs. Male partners are rarely invited to participate in ANCs as some health professionals persist in excluding the partner from care and services offered to pregnant women.

For my husband, a man should not meddle in women’s affairs. Maternal health is the business of women. If his wife has a problem, he will have no problem to accompany her; otherwise, we can let the woman manage her pregnancy. (pregnant woman 03)

The fear of Haitian men to go to the hospital was a significant obstacle to male involvement in PMTCT. According to the respondents, men go to the hospital only when they are sick. In most cases, they are stoic in the face of disease. Going to the hospital at the request of his partner is unlikely for the man. “Usually, my partner goes to the hospital when he is sick. If he is not sick, he has no obligation to go to the hospital” (pregnant woman 01).

Male partners were not satisfied with the services provided during prenatal visits. According to most respondents (*n* = 5), men felt ostracized or unwelcome in health facilities. According to them, care services were an inappropriate framework for men’s expectations for reasons related to hours of service incompatible with their normal daily activities and long waiting periods for the ANC:

My husband works six days a week. Most of the time, he leaves at 6 am and returns late in the evening. PMTCT services are closed on weekends and at 4 pm during the week. My husband goes to work instead of coming to the hospital with me. (pregnant woman 02)

The nurse site manager corroborated this information as being a barrier to male partner involvement in PMTCT services.

According to my partner, wait times are unacceptable. For him, it’s a waste of time. (pregnant woman 03)

Health centers are unsuitable for receiving men. Doctors are not welcoming. (pregnant woman 05)

Our quantitative and qualitative findings are complementary and mostly concordant regarding male partners’ involvement in PMTCT and associated factors. For example, in quantitative findings, only a quarter of male partners accompanied their women to the ANC. This result is consistent with some barriers to male involvement, such as gender relations and organizational of antenatal services. We have not identified any divergent results. Complementary and concordant findings from qualitative and quantitative data deserve further consideration.

## Discussion

### Summary

This study aimed to determine the level of involvement of partners of pregnant women living with HIV in PMTCT services and associated factors and identify barriers to their involvement in PMTCT. A little under half of the male partners had a high level of involvement. Factors associated with male involvement in PMTCT were being married and sharing HIV status with the male partner. Male partners with a positive HIV status were more involved in PMTCT. Barriers to male involvement in PMTCT included the conflict between opening hours of the ANC and the male partner’s schedule, waiting time at the ANC, and the perception of antenatal care as being women business.

### Comparison with Existing Literature

The results of this study revealed that, according to HIV-positive pregnant women, more than half of their male partners (51%) had a high level of PMTCT involvement. While this level of involvement is limited, it is higher than that reported in other studies conducted in Africa. A cross-sectional study conducted in eastern Uganda suggested that 26% of male partners were involved in PMTCT (Byamugisha et al., 2010). The level of involvement was even lower in Gondar’s study in northwestern Ethiopia, where Amano and Musa (2016) reported that the involvement of male partners in PMTCT was 20%. The difference observed between the studies could be attributed to the various sociocultural and economic contexts that persist in these countries.

When looking at the different dimensions and barriers of male involvement, the results are more mixed. Support of pregnant women by male partners during ANC visits was low (25%). Interviewed women reported men’s lack of time to come to ANC visits as a barrier to PMTCT involvement (Yeganeh et al., 2017). These findings are consistent with other studies conducted elsewhere in sub-Saharan Africa (Ditekemena et al., 2012), and particularly in Uganda (Byamugisha et al., 2010) and Ethiopia (Amano & Musa, 2016) in which 5% and 27% of men, respectively, accompanied their pregnant women to the ANC. A possible explanation of these results is the inadequacy of the ANC schedules, which are not compatible with the partners’ work schedules (Yeganeh et al., 2017). Other male involvement barriers identified by our participants are men’s beliefs that antenatal care and maternal health services are only women’s affairs and the lack of friendliness of these services toward men. Several previous studies have also reported that the long waiting periods and the poor reception they receive constitute barriers to the accompaniment of their spouses at ANCs (Amano & Musa, 2016; Kalembo et al., 2013). In our study, 90% of pregnant women reported receiving financial support from their partners to attend the ANC. These results were comparable to other studies in Uganda (97.5%) (Byamugisha et al., 2010) and Ethiopia (87.5%) (Abuhay, 2014). The Ethiopian research reported that male partners who have a monthly salary or are self-employed with a stable financial income are more likely to accompany their wives during antenatal consultations (Abuhay, 2014). A retrospective study conducted in Malawi presented similar results (Kalembo et al., 2013). These findings are also supported by studies conducted in Western Kenya (Oyugi et al., 2017), rural South Africa (Matseke et al., 2017), Tanzania (Elias et al., 2017), and a systematic review carried out in sub-Saharan Africa (Ditekemena et al., 2012). The explanation of these results could be the man’s perception as the one who provides money for the family’s subsistence and medical care (Morfaw et al., 2013). A qualitative analysis showed that men preferred to be satisfied with their financial obligations rather than worry about their presence in the ANC or share responsibilities as part of being a couple during and after pregnancy.

Our results revealed that being married, sharing HIV status with the partner, and knowledge of the partner’s HIV status are the factors associated with men’s male involvement in PMTCT activities. Similar studies in Uganda (Byamugisha et al., 2010) and South Africa (Matseke et al., 2017) have reported the same results. Mutual disclosure of HIV status among partners is essential as it allows decision-making regarding healthy antenatal and postnatal care choices (Ekama et al., 2012; Kirsten et al., 2011; Walcott et al., 2013). Disclosure of HIV positive status by women can encourage their partners to learn about reproductive health options (Ekama et al., 2012; Kirsten et al., 2011; Walcott et al., 2013). Male partner involvement interventions that support mutual HIV testing and disclosure among both partners would thus be beneficial in this regard (Ekama et al., 2012; Kirsten et al., 2011; Walcott et al., 2013). Failure to consider the HIV status of the partner of pregnant women may limit the effect of ART to prevent MTCT and represent a risk factor for superinfection of pregnant women with a resistant virus if their partner is living with HIV and does not benefit from ART (Suksomboon et al., 2007). Thus, health authorities should encourage HIV testing among partners of HIV-infected people.

### Implications for Research and Clinical Practice

This research work opens an avenue for further research and programs rebuilding in reproductive health in the Haitian context. Our results could offer the opportunity to look at gender dynamics, culture, and men’s role as determinants of maternal and child health.

Indeed, studies conducted in Africa have reported that gender equality, shared decision-making in the household, education, and improving women’s socioeconomic conditions could improve mother and child health (Lowe et al., 2016; Manda-Taylor et al., 2017; Singh et al., 2015). To facilitate male involvement, it would be imperative to change maternal and child health programs and services toward family services that are more inclusive, less limiting, and take into account men’s needs (Bougangue & Ling, 2017; Morgan et al., 2017).

### Strengths and Limitations of the Study

To the best of our knowledge, this study is the first to provide data on men’s involvement in PMTCT in Haiti using both quantitative and qualitative methods. This is one of the few studies that only surveyed HIV-infected pregnant women to determine male partners’ level of involvement in PMTCT. In most of the other studies identified, many respondents were HIV negative. We adhered to the STROBE guidelines (von Elm et al., 2008) and the consolidated criteria for reporting qualitative research (COREQ) guidelines (Tong et al., 2007).

There are potential limitations to this study. First, it is possible that pregnant women who agreed to participate had a better experience regarding their partners’ involvement in PMTCT than those who refused to participate. Second, it is difficult to avoid the possibility of a social desirability bias. Third, this study included convenience sampling that raises a concern of selection bias and generalization to the whole population targeted by this study. Fourth, this study presents male involvement from the female perspective and not the male. We cannot be sure whether the women presented the facts as they are or their assumptions. Finally, due to time and resources constraints, we only recruited 102 women for the quantitative part and interviewed six women for the qualitative part. Thus, data saturation was not achieved. The small sample size could also affect the generalization of findings.

### Conclusion

This study found that just more than half of male partners had a high PMTCT involvement index, as reported from a sample of HIV-positive women in Haiti. The participation of partners in ANC visits and constant condom use during pregnancy in the couple is where men are the least involved. Male involvement in the whole PMTCT cascade is necessary to achieve more significant results. Several barriers related to gender relations, cultural beliefs, and care organization may hinder this involvement. Thus, health authorities need to put culturally accepted and appropriate strategies to improve pregnant women’s partners in PMTCT and sensitize them on their essential role in the reduction of MTCT in Haiti.
